# Tripartite Motif 22 and Class II Transactivator Restriction Factors: Unveiling Their Concerted Action against Retroviruses

**DOI:** 10.3389/fimmu.2017.01362

**Published:** 2017-10-18

**Authors:** Greta Forlani, Roberto S. Accolla

**Affiliations:** ^1^Laboratories of General Pathology and Immunology “Giovanna Tosi”, Department of Medicine and Surgery, University of Insubria, Varese, Italy

**Keywords:** restriction factors, tripartite motif 22, class II transactivator, promyleocitic leukemia protein, CyclinT1, nuclear bodies, human immunodeficiency virus 1

## Abstract

Coevolution of the three basic mechanisms of immunity, intrinsic, innate and adaptive, is a constant feature of the host defense against pathogens. Within this frame, a peculiar role is played by restriction factors (RFs), elements of intrinsic immunity that interfere with viral life cycle. Often considered as molecules whose specific functions are distinct and unrelated among themselves recent results indicate instead, at least for some of them, a concerted action against the pathogen. Here we review recent findings on the antiviral activity of tripartite motif 22 (TRIM22) and class II transactivator (CIITA), first discovered as human immunodeficiency virus 1 RFs, but endowed with general antiviral activity. TRIM22 and CIITA provide the first example of cellular proteins acting together to potentiate their intrinsic immunity.

## Restriction Factors (RFs): Key Elements of Intrinsic Immunity Against Viruses

**Figure 1:**
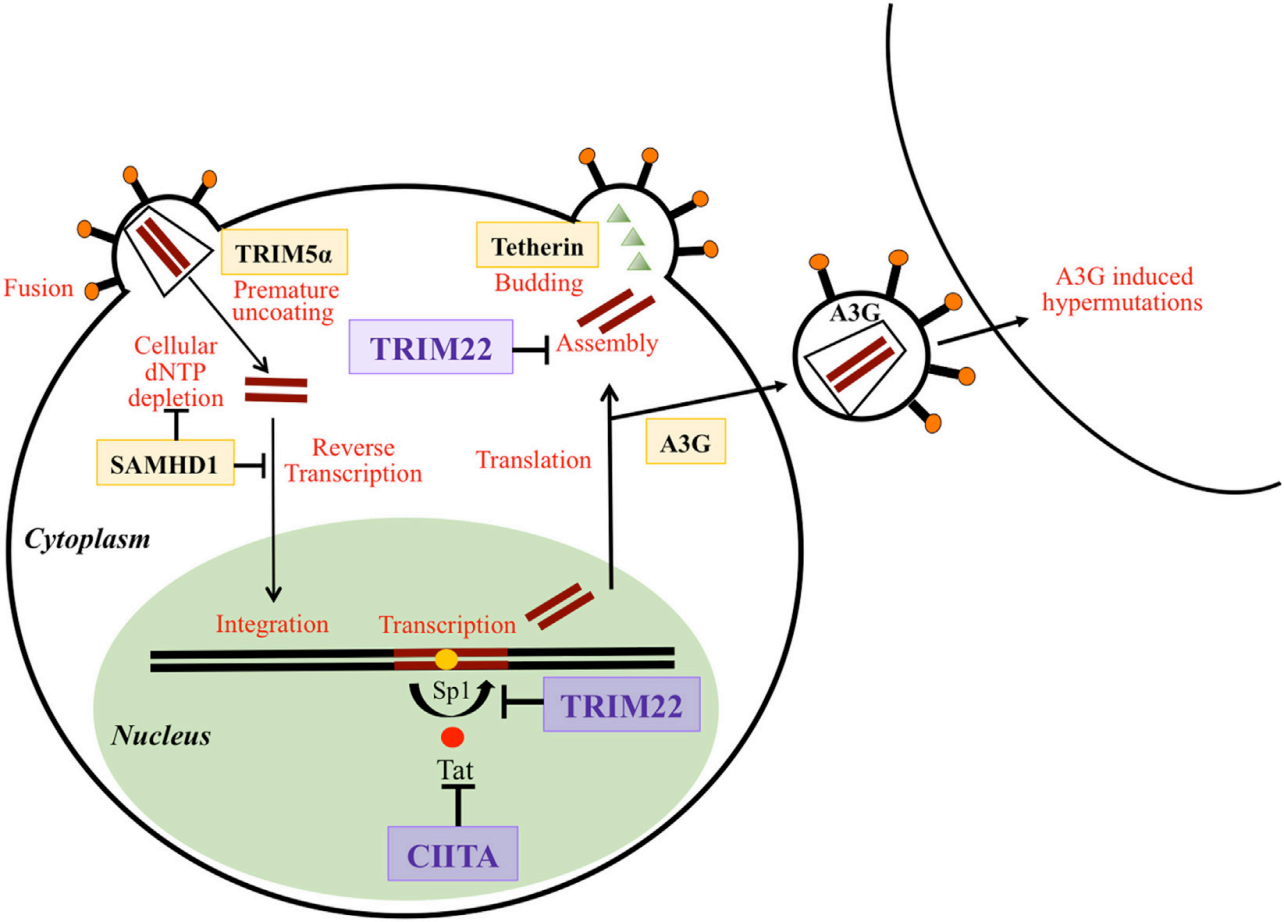
Schematic overview of the action of different restriction factors (RFs) at various stages of the human immunodeficiency virus life cycle. RFs are in bold and framed. Their targeted functions on the virus life cycle are indicated in red. Tripartite motif 22 (TRIM22) and class II transactivator (CIITA), the two RFs described in this review are magnified and written in violet. TRIM5α acts at early phases of virus infection by disturbing and inducing a premature uncoating. SAMHD1 inhibits reverse transcription of the viral RNA by either targeting the dNTP pool or reverse transcription products. CIITA acts by inhibiting the Tat-mediated activation of transcription and particularly the elongation of primary transcripts. TRIM22 acts both at the level of transcription of viral DNA by blocking the action of Sp1 and at the level of assembly of new viral particles. Tetherin blocks the budding of viral particles at the cell surface. APOBEC3G (A3G) incorporates into newly formed viral particles and after infection of a new cell A3G induces hypermutations during the process of reverse transcription thus inhibiting the viral replication cycle.

In recent years, the role of viral RFs as potent effectors of intrinsic antiviral immunity has become more clear (1–5). Most studies have focused on the mechanisms of host-mediated restriction of human immunodeficiency virus 1 (HIV-1), in which RFs exert intrinsic antiviral activity by targeting different steps of the HIV-1 life cycle, from capsid uncoating to viral budding (4, 6–11). Although RFs can be expressed constitutively in the host cell, most of them are potently upregulated by molecules of innate immunity such as type I and type II interferons (IFN), reinforcing the concept that they are crucial players of the immune defense against retroviruses (12). The most extensively studied RFs are Apolipoprotein B mRNA-editing enzyme catalytic polypeptide-like 3G (APOBEC3G), Tripartite motif 5-alpha (TRIM5α), tetherin (also known as BST-2, CD317, or HM1.24), and Sterile alpha motif domain and histidine aspartic domain containing protein1 (SAMHD1) (Figure 1).

To evade host restriction, HIV-1 has developed some countermeasures by using viral accessory proteins or by inducing specific mutations on protein interfaces (13). For example, the human APOBEC3 G/F enzymes are cytidine deaminases packaged into the virus particles during assembly. They inhibit reverse transcription by deaminating viral cDNA cytosines to uracils, thus introducing G-to-A hypermutations in the viral genome (14–17). The viral infectivity factor of HIV-1 counteracts the APOBEC3 activity, by mediating its proteasomal degradation. Similar to APOBEC3 enzymes SAMHD1 also inhibits retroviral transcription, by depleting the intracellular pool of deoxynucleoside triphosphates available during early reverse transcription (17). SAMHD1 activity is prevented in HIV-2 and related SIVs by the viral proteins Vpx and Vpr (18, 19). Different from the host factors mentioned above, TRIM5α acts as a species-specific antiviral factor. Indeed rhesus monkey TRIM5α (rhTRIM5α), but not human TRIM5α, potently limits HIV-1 infection in Old World monkeys by targeting the viral capsid, thus preventing the uncoating of the viral pre-integration complex (20). Tetherin targets the post-integration stages of HIV-1 replication and prevents the release of nascent viral particles by anchoring virions on the cell surface of HIV-1 infected cells. Viral proteins Vpu and Env overcome tetherin restriction by sequestering tetherin in cellular compartments away from sites of viral budding or by targeting it for degradation into lysosomes (21).

Often considered as independent elements in the action against virus infection, recent studies unveil, instead, that RFs may act in concert against the pathogen. This review will discuss the example of cooperativeness in HIV-1 restriction of two recently described RFs, the tripartite motif 22 (TRIM22) and the MHC class II transactivator (CIITA). Importantly, this functional synergy is mirrored by their cellular policy of getting together in common subcellular compartments where other crucial factors controlling HIV-1 replication convene. Thus, TRIM22 and CIITA represent a clear example of concerted action whose final goal is favored by compartmentalization of multiple players involved in fighting against HIV-1.

## Mechanisms of TRIM22 and CIITA Viral Restriction

Tripartite motif 22 was first identified as an IFN-inducible protein that restricts HIV-1 transcription (Figure 1) (22). The first clinical evidence highlighting the antiviral function of TRIM22 *in vivo* was reported by Singh et al. demonstrating that in peripheral blood mononuclear cells (PBMCs) of infected patients, the expression of TRIM22 was significantly increased and correlated with lower viral loads and higher CD4+ T cell counts (23, 24). At present, several reports demonstrated that TRIM22 acts as RF against a broad spectrum of viruses. Besides inhibiting HIV-1 transcription, TRIM22 also inhibits Influenza A virus (25), Hepatitis B and C viruses (26, 27), and encephalomyocarditis virus (28), by using different mechanisms. TRIM22 belongs to TRIM family of proteins exerting various functions, including cellular proliferation, apoptosis, oncogenesis, and antiviral activity (29). TRIM proteins are characterized by the RBCC motif, consisting in a Really Interesting New Gene (RING) domain, one or two B-boxes followed by a coiled-coil (CC) region. The C terminal part is specific for each TRIM (6). The RING domain is involved in protein–protein interactions and is associated with E3 ubiquitin ligase activity (30). The CC region is crucial for the formation of protein complexes and promotes homo- and hetero-oligomerization that may cause dislocation in distinct subcellular compartments (31, 32). TRIM22, as other TRIM proteins, contains a C-terminal B30.2/SPRY domain, whose function has not been fully clarified. Some studies have shown that it is also critical for TRIM22 nuclear localization and formation of nuclear bodies (33). Furthermore, the B30.2/SPRY was shown to dictate the different subcellular distributions and thus specific functions of TRIM family members (34, 35). Indeed, the B30.2/SPRY domain is essential for TRIM22-mediated activation of nuclear factor kappa B (NF-κB) (26, 36) and possibly for the recently described TRIM22-mediated monocyte apoptosis (37). The intimate reasons of the distinct subcellular distribution of TRIM22 found in various studies, however, remain controversial. Indeed, several factors may influence the subcellular localization of the protein, such as the assessment of endogenous versus exogenous expression, the cell line analyzed, the cell cycle, being epitope-tagged or untagged, and the method of fixation used in the analysis (32). We and others have reported that TRIM22 localizes in the nucleus as punctate bodies (26, 38, 39). In some cells, these TRIM22 nuclear bodies partially overlap with Cajal bodies (34, 35) or centrosome (39). Other investigators have shown that TRIM22 may also localize in the cytoplasm with a diffuse and/or a speckled pattern (33–35). Here, TRIM22 localized in vimentin-containing structures (39).

The considerations expressed above are crucial for a precise delineation of additional mechanisms of inhibition of HIV-1 replication mediated by TRIM22. Indeed, Barr et al. reported that cytoplasmic TRIM22 ectopically expressed in human epithelial HeLa or in osteosarcoma HOS-CD4-CXCR4 cell lines blocked the release of HIV-1 particles by targeting Gag and thus inhibiting HIV particle assembly (Figure 1). This inhibitory function depended on its RING domain (40). Other reports demonstrated that TRIM22 overexpressed in COS-1, human macrophages or in 293 T cells impaired basal as well as phorbol-12-myristate13-acetate (PMA)-ionomycin induced HIV-1 long terminal repeat (LTR)-mediated transcription when present in the nucleus (7, 22, 41). TRIM22 suppressed transcription from HIV-1 LTR independent of its E3 ubiquitin ligase activity and did not inhibit neither Tat nor NF-kB-activated HIV-1 transcription (7). Recently, this effect has been attributed to the capacity of TRIM22 to affect the binding of Specific protein 1 (Sp1) to the HIV-1 LTR promoter region (42) (Figure 1). Although most of the studies demonstrating the antiviral function of TRIM22 have been conducted by using ectopically expressed proteins, some reports indicated that physiologic levels of TRIM22 could indeed interfere with HIV-1 replication (7, 24). In particular, Kajaste-Rudnitski et al. identified TRIM22 as the HIV-1 RF expressed in a subset of U937 promonocytic cell clones poorly permissive to HIV-1 replication, and not expressed in the isogenic HIV-1 permissive U937 cell clones (7, 43). In this regard, they found that the depletion of TRIM22 in non-permissive U937 clones increased viral LTR transcription to levels closer to those observed in the permissive cells, thus suggesting that TRIM22 contributed to HIV-1 refractory phenotype of poorly permissive cells (7). Consistently, exogenous expression of TRIM22 in permissive clones reduced HIV-1 LTR transcription.

The MHC class II transcriptional activator, also designed CIITA, was originally discovered as a master regulator of major histocompatibility complex (MHC) class II gene expression (44–46). Both constitutive and IFNγ-inducible expression of MHC-II are under the control of CIITA (47). By regulating the expression of all MHC class II genes, CIITA controls antigen presentation to CD4+ T helper (TH) cells, thus playing a critical role in the triggering of the adaptive immune response against a wide variety of antigens including pathogens (48, 49) and tumors (50). CIITA is a large protein characterized by distinct functional domains critical for its transactivating function: the N-terminal transcription activation domain (AD); the proline/serine/threonine-rich region (P/S/T); the GTP-binding domain (GBD), and the C-terminal leucine-rich repeats (LRR) important for the subcellular localization of the protein (51–53).

Besides its role in antigen presentation, via the regulation of MHC-II genes expression, it was demonstrated that CIITA restricts HIV-1 infection in human T cells by acting at the level of viral LTR transcription (Figure 1). CIITA binds to CyclinT1 of the positive transcription elongation factor b (P-TEFb) (54) and competes with the viral transactivator Tat for the binding to CyclinT1 (55). Subsequent studies have shown that CIITA acts as a general RF against retroviruses (49). Indeed, CIITA inhibits human T lymphotropic virus 2 (HTLV-2) replication, by targeting the viral transactivator Tax-2 (56). This inhibition occurs by preventing the association of Tax-2 to the common binding element nuclear factor Y (NF-Y), used by the virus to promote viral transcription (57, 58). Importantly, CIITA blocks the replication of the HTLV-1, the first discovered human oncogenic retrovirus (59), responsible for a severe form of T cell leukemia-lymphoma of the adult (ATL). Here, CIITA exerts a double function: it competes with the viral transactivator Tax-1 for binding with key cellular factors required for Tax-1 transactivating function on the viral LTR, and it binds directly to the viral transactivator, greatly limiting its intracellular migration (49, 60). Thus, CIITA restricts HTLV-1 viral replication by physically and functionally excluding Tax-1 transactivator from its crucial action in recruiting cellular transcription factors on virus LTR promoter to initiate viral replication. Tax-1 is crucial not only for the regulation of viral replication but also for its key action on cellular transformation predisposing to ATL. In this regard, it has been recently demonstrated that CIITA affects not only Tax-1 transactivating capacity but also Tax-1-mediated NF-kB activation, the crucial molecular event in initiation of leukemogenesis (61). Here, CIITA exerts its inhibitory function mainly by retaining Tax-1-NF-kB complex in the cytoplasm, thus preventing the translocation of NF-kB to the nucleus and the consequent activation of NF-kB responsive genes. In this regard, CIITA may counteract the oncogenic potential of HTLV-1 (52, 53).

## The Importance of Subcellular Compartmentalization to Counteract Viral Replication

As mentioned above, the first clue that CIITA may act as an RF was made when it was found that CIITA inhibited HIV-1 replication in T cells (55). Relevant to HIV infection, subsequent recent studies have shown that CIITA, like TRIM22, was expressed in HIV-1 poorly permissive U937 myeloid cell clones, and absent in the permissive U937 myeloid parental cells (52, 53). Importantly, transfection of CIITA in HIV-1 permissive U937 clones resulted in the inhibition of Tat-dependent HIV-1 replication independent of TRIM22 (52, 53). Thus, CIITA is an HIV-1 RF for both lymphoid and myeloid cells.

**Figure 2:**
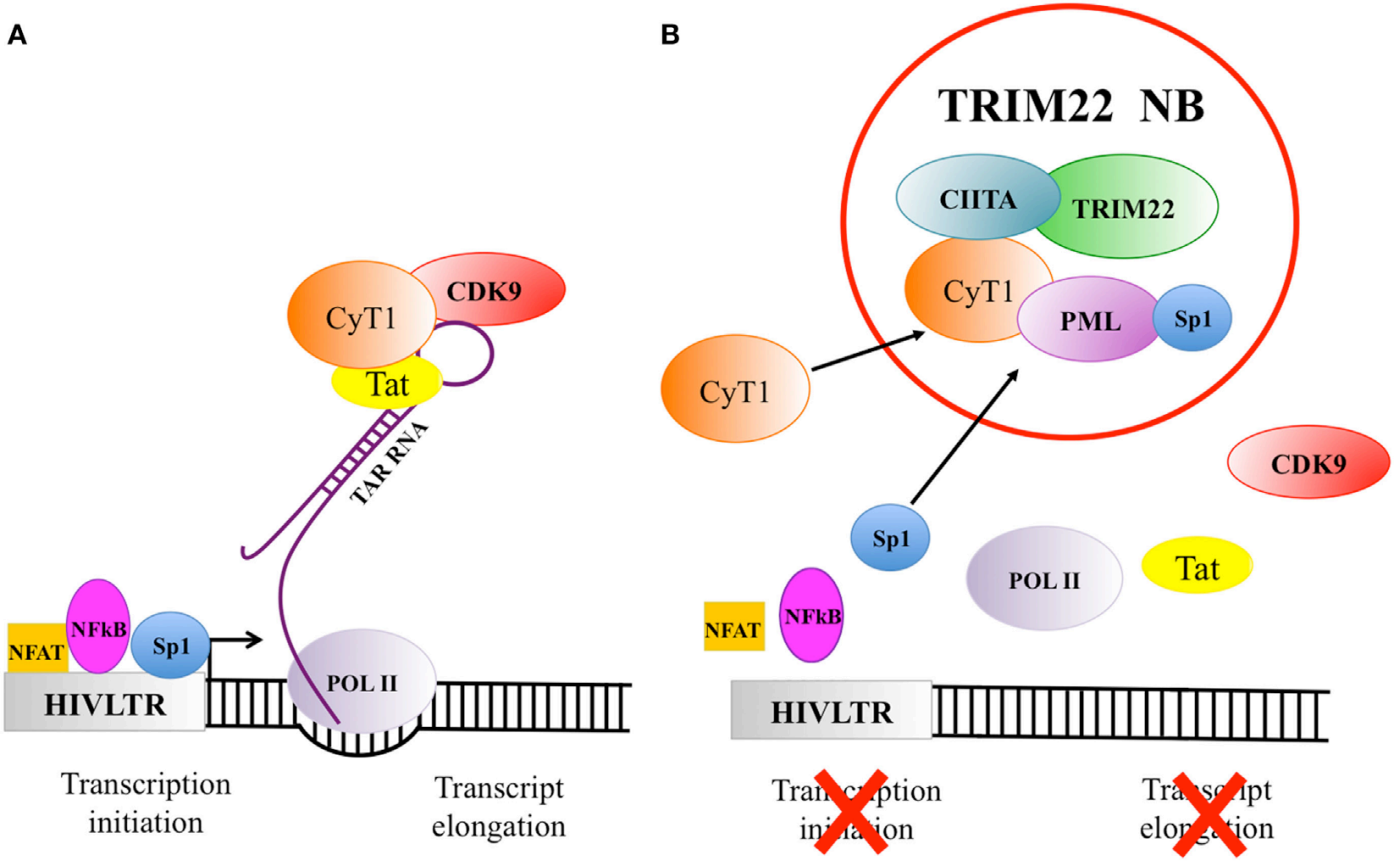
The recruitment of factors in tripartite motif 22 (TRIM22) nuclear bodies strongly inhibits viral gene expression. **(A)** Human immunodeficiency virus 1 (HIV-1) gene transcription and expression are essential steps in the viral life cycle. HIV-1 transcription is under the control of several factors among which NFAT, NFkB, and Sp1 (symbolized in the figure as a square, oval and circle, respectively) are particularly important. They bind to specific sequences of the 5′LTR promoter/enhancer region to activate viral genome transcription. The cellular transcription factor Sp1 is one of the most potent inducer of HIV-1 gene expression and it is crucial to initiate the basal transcription of viral RNA. The viral transactivator protein Tat plays a central role in sustaining a high level of HIV-1 replication. When Tat is present and binds to the bulge of the Trans-Activation Response (TAR) RNA element, the cellular transcription elongation factor P-TEFb, composed by the regulatory subunit CyclinT1 (CyT1) and the kinase subunit CDK9, will be recruited. Upon phosphorylation of RNA polymerase II (POL II), TAR can be elongated to the full length viral RNA. **(B)** TRIM22 nuclear bodies, containing class II transactivator (CIITA) and PML, recruit also CyT1 and Sp1, thus impairing both HIV gene transcription initiation and transcription elongation.

It was apparent, however, that neither CIITA nor TRIM22 alone could restore completely the level of HIV-1 inhibition of replication observed in poorly permissive cells (7, 52, 53), suggesting that the simultaneous expression of these two RF could be required for a more effective HIV-1 restriction (52, 53). To better delineate the biological basis of the possible cooperativeness between CIITA and TRIM22, further experiments were designed to assess possible interaction and subcellular localization of the two RFs. It was found that TRIM22 interacted with CIITA and recruited it in newly defined compartments that we designated as TRIM22 nuclear bodies (Figure 2) (38). Interestingly TRIM19, another member of TRIM family, also known as promyleocitic leukemia protein (PML), and reported to inhibit the replication of various viruses, including HIV-1 (62–66), co-localized to a significant number of TRIM22 bodies. Relevant to this point, previous studies showed that PML and CIITA homed to the same nuclear bodies when cells were treated with IFN-γ (67). Thus, it was important to assess whether in cells co-expressing TRIM22 and CIITA, the latter was recruited by TRIM22 in nuclear bodies containing also endogenous PML. Indeed, this was the case (38). Of further relevance was the fact that TRIM22 nuclear bodies hosted also CyclinT1 of the P-TEFb complex, a key component of the trascription machinery used by HIV-1 to promote viral gene expression. CyclinT1 was previously shown not only to be bound by CIITA to inhibit Tat-mediated HIV-1 transcription (55), but also to localize in PML bodies where aggregation with PML negatively affected Tat-induced LTR transcription (68). Interestingly, silenced but transcriptionally competent HIV-1 proviruses were shown to reside in close proximity to PML NBs and this association inhibited HIV-1 gene expression (69).

Taken together, these recent observations strongly point to the possibility that CIITA and PML cooperate in the inhibition of Tat-mediated HIV-1 LTR transactivation by competing with Tat for the binding to CyclinT1. Moreover, TRIM22 inhibits basal HIV-1 LTR transcription by affecting the binding of Sp1 to the viral promoter (42), and PML interacts with and sequesters Sp1 in PML nuclear bodies, suppressing the transcriptional function of Sp1 to its target sequence (70). Thus it is also possible that PML, by associating with Sp1, contributes to TRIM22-mediated inhibition of basal HIV-1 transcription as well (Figure 2). Within this frame, TRIM22 nuclear bodies may then be seen as the first example of an intracellular hub where several RFs may convene and act in concert to inhibit HIV-1 viral replication. This may be relevant for the mechanism of latency and persistence as the regulation of HIV-1 transcription might correlate not only with the localization of proviral DNA (69) but also with the recruitment-dependent synergy of key transcription factors with inhibitory function on viral transcription.

## Concluding Remarks and Future Perspective

Although several studies have focused at clarifying the antiviral mechanisms of host RFs, much research is still required particularly on those aspects related to the possible cooperative action of RF during viral infection. In this review, we summarized the recent acquisition on the function of two newly defined RFs, CIITA and TRIM22, against retroviruses, particularly HIV-1, highlighting peculiar aspects that strongly suggest the existence of a concerted action against HIV-1.

First, the generation of specific TRIM22 nuclear bodies and the accumulation of TRIM22 and CIITA in these compartments.

Second, the recruitment of CIITA and CyclinT1, a key component in the HIV-1 Tat-mediated elongation of HIV-1 primary transcripts, in the same TRIM22 nuclear bodies, an event that may ensure a potent inhibition of HIV-1 transcription by acting at basal (through TRIM22) and Tat-promoted (through CIITA) LTR transcription.

Third, the co-localization in TRIM22 nuclear bodies of PML, previously shown to affect Tat-dependent LTR transcription and to bind Sp1, can synergize with the inhibitory action of both CIITA and TRIM22.

The above findings now open up the field to novel investigations and additional interesting new questions related, for example, to the intimate molecular mechanisms that drive distinct RFs to migrate to the same endocellular compartments and to retain them in the endocellular compartment. Additionally, are these mechanisms of co-localization of molecules with potential synergic action against the virus an active response of the infected cells to the insult or, paradoxically, are used by the virus to tentatively counteract or even neutralize its intracellular opponents? In the latter case may RFs be used by the virus to favor a state of latency by reducing its capacity to replicate? Is the co-localization of several RFs in the same compartments the mirror of a recently acquired evolutionary action to better fight virus infection, or a mechanism to serve other diverse functions in cell homeostasis? CIITA is one of the most important regulators of adaptive immunity through its activating function on the expression of MHC class II genes and thus on antigen presentation to CD4+ TH cells. Is its function as RF against retroviruses a recent diversification of its duties or an old function preceeding its role on adaptive immunity?

Future research on the mechanisms of subcellular localization and redistribution as well as functional cooperativity between distinct RFs will certainly enlarge our knowledge on the complexity of host cell–pathogen interaction and provide new ideas and strategies to better counteract viral infectivity and spreading.

## Author Contributions

GF and RA participated in the conception and design of the review; revised the manuscript; read, critiqued, and approved the final manuscript.

## Conflict of Interest Statement

The authors declare that the research was conducted in the absence of any commercial or financial relationships that could be construed as a potential conflict of interest.
